# Environment, subsistence strategies and settlement seasonality in the Negev Highlands (Israel) during the Bronze and Iron Ages: The palynological evidence

**DOI:** 10.1371/journal.pone.0285358

**Published:** 2023-05-24

**Authors:** Dafna Langgut, Israel Finkelstein

**Affiliations:** 1 The Laboratory of Archaeobotany and Ancient Environments, Institute of Archaeology, and the Steinhardt Museum of Natural History, Tel Aviv University, Tel Aviv, Israel; 2 School of Archaeology and Maritime Cultures, University of Haifa, Haifa, Israel; Austrian Academy of Sciences, AUSTRIA

## Abstract

The Negev Highlands arid region (southern Levant) shows evidence of sharp settlement fluctuations, with several periods of strong human activity separated by centuries with no evidence of sedentary life. In this study, we used the palynological method in order to shed light on the region’s demographic history in the Bronze and Iron Ages. Fifty-four samples of pollen were collected and analyzed from secure archaeological contexts in four Negev Highlands sites: Nahal Boqer 66, dated to the Early Bronze Age and Early Intermediate Bronze Age (ca. 3200–2200 BCE); Ein Ziq, dated to the Early Intermediate Bronze Age (ca. 2500–2200 BCE); Mashabe Sade, dated to the Intermediate Bronze Age (ca. 2500–2000 BCE); and Haroa, dated to the Iron Age IIA (ca. late 10th through 9th centuries BCE). Our study revealed no evidence of cereal cultivation, with some hints that the inhabitants’ diets may have included plants gathered from the wild. Only one of the sites, Nahal Boqer 66, showed micro-indicators of animal dung remains, suggesting that the inhabitants herded animals. The palynological evidence did, however, emphasize that the livestock there were not fed or supplemented with agricultural by-products but rather grazed freely on wild vegetation. The pollen data also suggest that all four sites were occupied only during late winter and spring. The activity in the Negev Highlands during the third millennium BCE was probably related to the copper industry in the Arabah and to copper transportation to settled neighboring lands, especially Egypt. A relatively humid climate supported the trade through the Negev Highlands. Deterioration in both climate conditions and settlement activity was documented in the second half of the Intermediate Bronze Age.

## 1. Introduction

The area of the Negev Highlands in Israel is part of the arid zone of the southern Levant. As a consequence of favorable environmental conditions relative to the areas around it, this region features a dense system of archaeological remains. However, not all periods are represented. Based on the findings of multiple excavations and surveys, there were three periods of intense activity during the Bronze and Iron Ages (ca. 3500–600 BCE), which left behind remains of settlements, farms, animal pens and water installations: The Early Bronze Age II (ca. 3000–2900 BCE; [1]), the Intermediate Bronze Age (ca. 2500–1950 BCE, also known as the Early Bronze Age IV; [1]) and the Iron Age IIA (ca. 940–780 BCE; [2]). Other periods in this era, such as the Middle and Late Bronze Ages (ca. 1950–1150 BCE), have almost no archaeological representation [3–5, 6 and references therein]. Recent studies based on ^14^C and optically stimulated luminescence (OSL) dating, have pointed to human activity in the southern Levant arid regions during the Early Bronze Age IB and the Early Bronze Age III as well, documenting human presence in the region from an early stage of the Early Bronze Age through the first half of the Intermediate Bronze Age (ca. 3200–2200 BCE; [7–9]).

Past studies proposed that the Negev people subsisted on animal husbandry and opportunistic dry farming. Herding was deduced from stone structures interpreted as animal pens, and agriculture from the occurrence of grinding stones and sickle blades, as well as by stone walls along ephemeral streams (e.g., wadi terraces; [6, 10–14]). Yet, recent investigations of phytolith assemblages in Negev Highland Bronze and Iron Age sites, provided no signs of cereal cultivation [8, 15–19].

**Fig 1:**
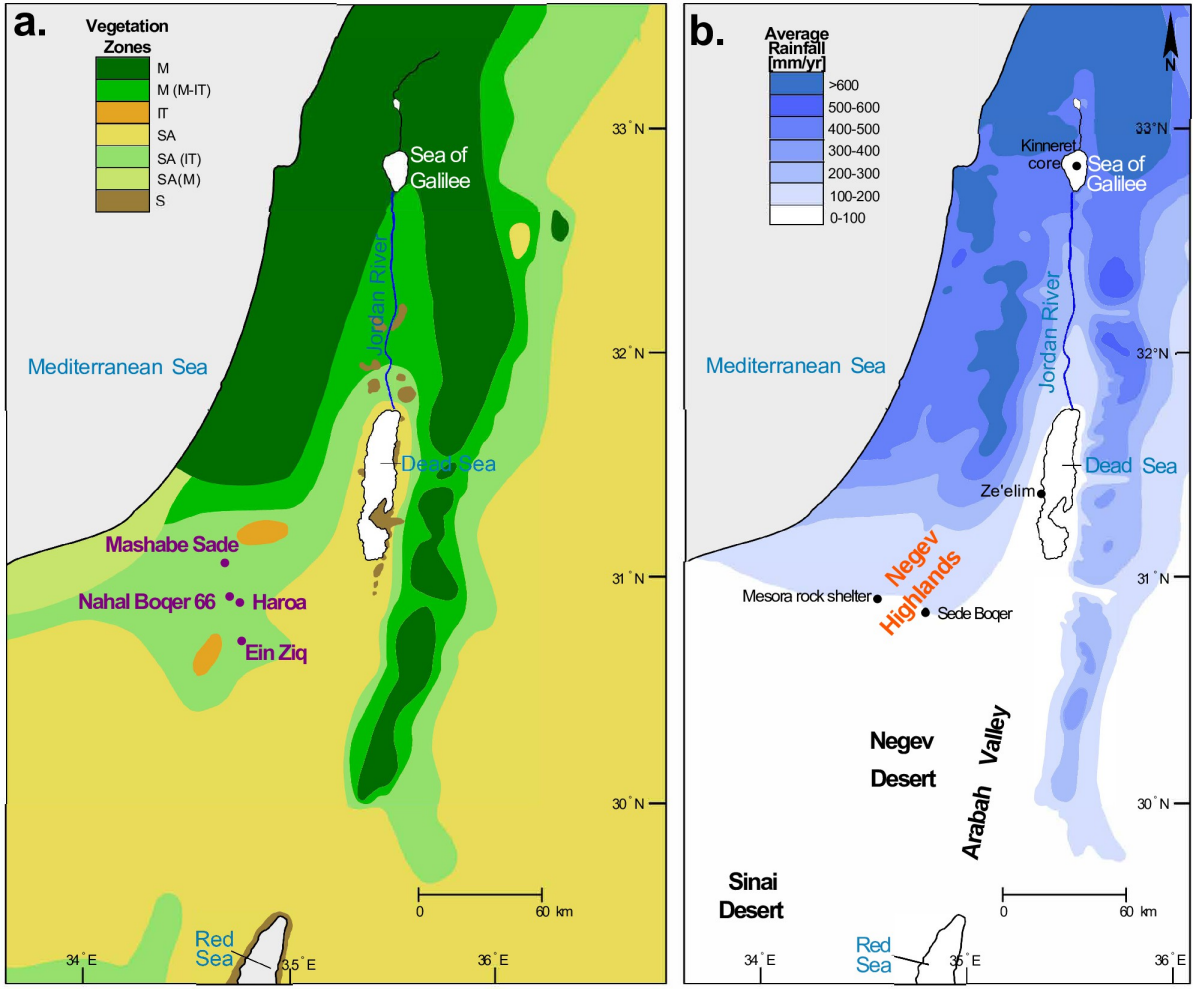
Phytogeographic and rainfall isohyet maps of the southern Levant indicating the investigated sites. (a) Distribution of phytogeographic zones in the southern Levant; M = Mediterranean zone (garrigue, maquis, woodland); IT = Irano-Turanian zone (steppe-land); SA = Saharo-Arabian zone (desert); S = Sudanian zone (penetration territory); (b) Map of the southern Levant indicating mean annual precipitation in mm. [Modified after 26: Fig 1). Prepared by M. Cavanagh.

Several factors have been suggested as the cause of the settlement fluctuations in the Negev Highlands. Because this area is part of the southern Levant desert environment (< 200 mm of rain annually, Fig 1), some scholars have linked these oscillations to climate changes [e.g., 3, 20]. According to a contrasting theory, these demographic oscillations are related to socio-economic opportunities and geo-political changes [6, 7, 9, 21, 22]. One example is the participation of local groups in copper production in the Arabah Valley and the transportation of copper to Egypt and other locales in the ancient Near East [7, 9, 23, 24]; another is the transportation of lucrative south Arabian commodities, such as myrrh and frankincense [e.g., 25]. Human pressure on the natural arid environment of the southern Levant has also been suggested as a factor that influenced the settlement abandonment process [26].

Three questions concerning the waves of settlements in the Negev Highlands remain unresolved:

1. Subsistence: Did the inhabitants of the Negev Highlands engage in agriculture? (currently under a scholarly debate).
2. Seasonality: Were the Negev Highlands sites inhabited year-round or just seasonally?
3. Vegetation and climate: What was the role of the environment in the demographic trends of the region?

As will be demonstrated in this study, our analysis of fossil pollen from several Negev Highlands sites allowed us to address these questions.

## 2. The Negev Highlands

### 2.1 Climate and environment

**Fig 2:**
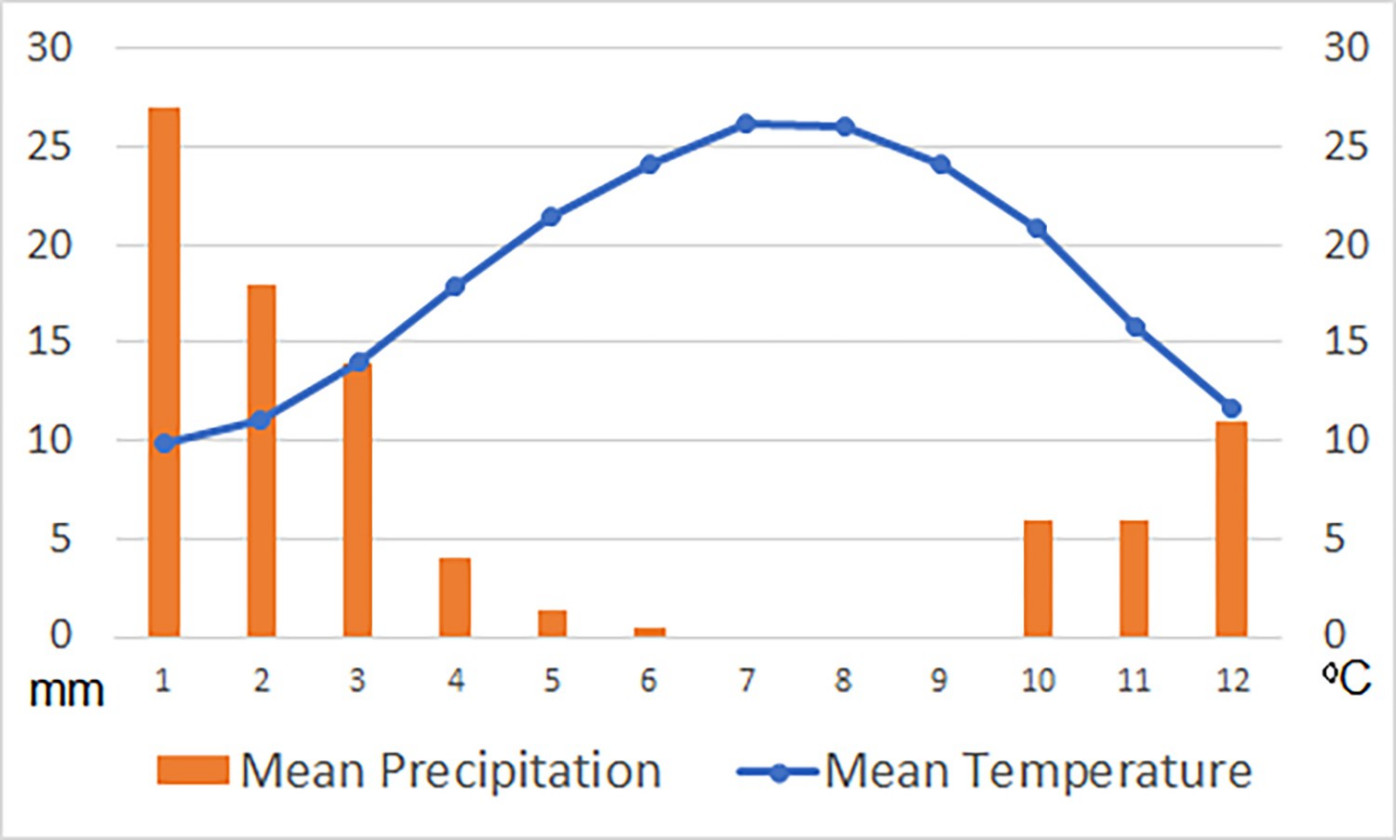
Sede Boqer weather station data. Average monthly temperature and precipitation for the period of 1991–2020. Data were downloaded from the Israel Meteorological Service (2023).

**Fig 3:**
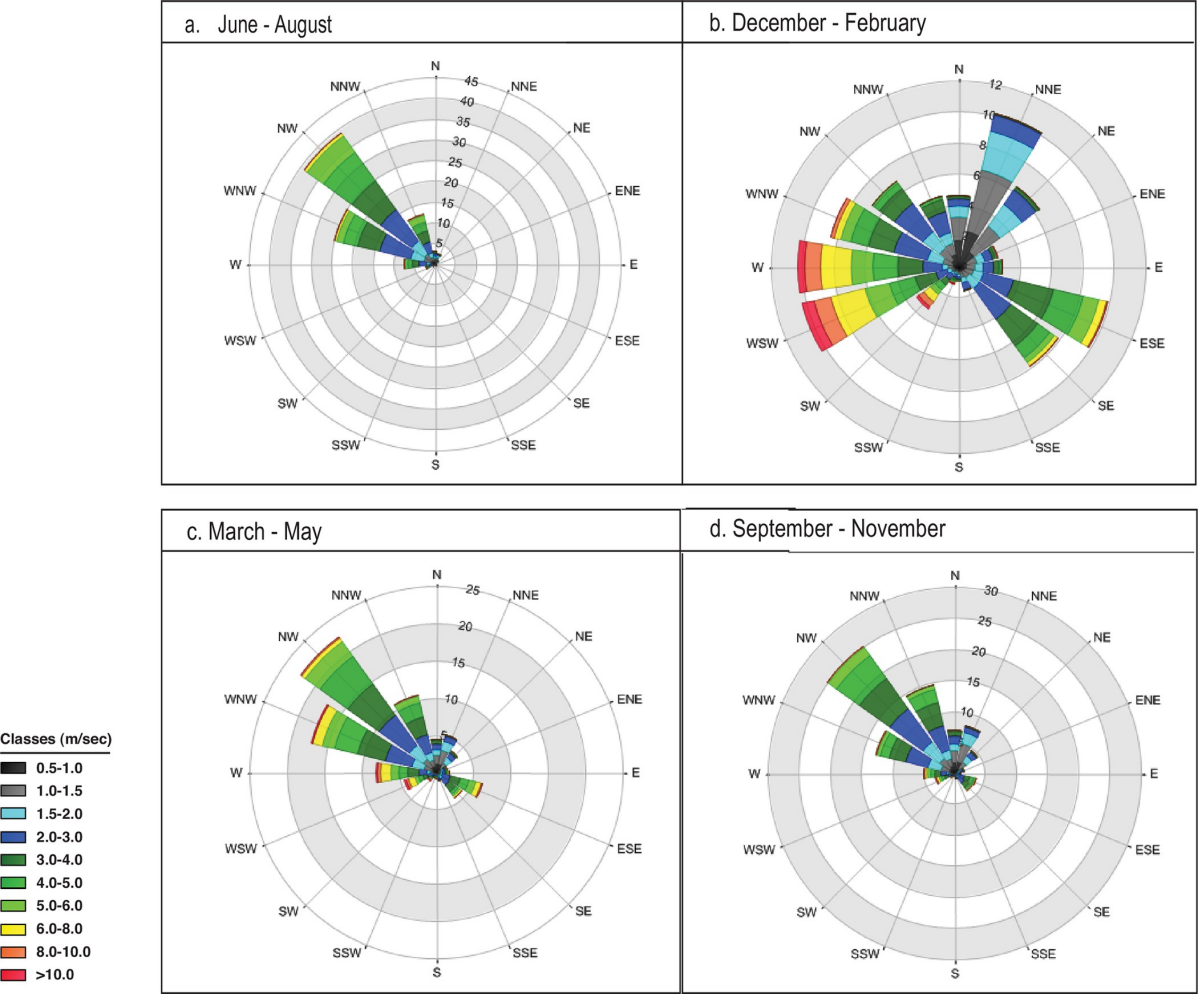
Average seasonal wind roses illustrating wind direction and speed (m/second) at Sede Boqer for the period 2003–2012. (a) Summer (June-August). (b) Winter (December-February). (c) Spring (March-May). (d) Autumn (September-November). Data were downloaded from the Israel Meteorological Service (2023).

The Negev Highlands area receives an average annual precipitation in the range of 100–200 mm (Fig 1; [27]), which usually falls from October to May (Fig 2). Summers are very hot, while winters are cold and windy. The dominant winds come from the northwest (Fig 3). Elevation ranges from ca. 400 m above sea level (asl) in the north to ca. 1,000 m asl in the south. The area is characterized by a set of parallel, northeast–southwest oriented anticlines and synclines, with broad loess-filled valleys between the folded ridges. The runoff that accumulates in these loessial wadis penetrates into the deeper soil layers and significantly increases the amount of water available for subsequent plant growth, creating microhabitats far more humid than the surrounding area [28]. Runoff water may also be found during winter as small potholes carved in the hard bedrock sometimes endure until the summer. The combination of higher elevation, relatively higher precipitation compared to areas surrounding it, and alluvial wadi beds with high water availability makes the region a favorable ecological niche for human exploitation in comparison to other areas in the arid regions of the southern Levant. In the alluvial wadi beds, seasonal opportunistic dry-farming (mainly barley) by Bedouins has been observed in recent decades.

The Negev Highlands is situated in a geographical transition zone between the Mediterranean climate to the north and the harsh arid conditions characterizing the rest of the area further to the south (Fig 1). Today the Atlantic–Mediterranean cyclones comprise the major source of rainfall in the northern and central Negev Desert (including the Negev Highlands), while in the southern Negev, a substantial part of the rainfall originates from the Red Sea Trough [29, 30]. This climate scenario was also prevalent during the second half of the Holocene [e.g., 31]. A dramatic north-to-south gradient of annual precipitation occurs between about 32°N (where annual rainfall is ca. 500 mm) and 31°N (< 100 mm; Fig 1). Such a large gradient cannot be found elsewhere in Mediterranean climates (except where orography controls the rainfall distribution; [29, 32]. As a result, even minor climatic fluctuations in northern Israel and Arabia/Sahara could alter the boundaries between the Mediterranean and desert zones, thus significantly affecting the status of the Negev Highlands transitional zone between them, and meaningfully changing the potential for human exploitation. Such minor climate changes may not even be archaeologically visible in the greener parts to the north or in the stable harsh desert to the south. The transition zone between the Mediterranean environment and the desert was therefore defined from a historical-archaeological perspective as the most sensitive climate-human links recorder in the Levantine region [33, 34]. Years of improved precipitation would push this transitional zone to the south and east, while dry years would drive it to the north and west [33]. Settlements situated near perennial sources of water may have managed to survive longer in periods of increasing aridity, whereas other sites may have been abandoned. This region was also recognized as the most vulnerable along the regional rainfall gradient (90 up to ca. 800 mm) in terms of grazing opportunities. It was found that herbaceous annual primary productivity is more affected by droughts and inter-annual rainfall variations than the mesic Mediterranean ecosystem [35]. This means that even a minor reduction in precipitation will significantly impact herding opportunities; this is not necessarily the case in the Mediterranean zone.

The shift of this semi-arid steppe zone is reflected in the regional pollen diagrams (Lake Kinneret [Sea of Galilee sediment core] and the Dead Sea [Ze’elim sediment outcrop]) by changes in the arboreal Mediterranean pollen values: decreasing percentages indicate the shrinkage of the natural Mediterranean maquis/forest and the shifting of the semi-arid boundaries to the north and west due to less available moisture; increasing values of the natural Mediterranean tree pollen marks the opposite.

Since there is no high-resolution paleoclimatological record that originated from the Negev area itself, we are using the pollen records from Lake Kinneret and the Dead Sea to describe the climate conditions during periods of occupation in the Negev Highlands [36–38]. These pollen records are sensitive to the conditions in both the Mediterranean area and the Irano-Turanian vegetation belt, as the two lakes collect wind-driven pollen from these two adjacent zones. In addition to airborne pollen, they receive stream-driven pollen mainly through the Jordan River, but also via local streams. Based on our Lake Kinneret and Dead Sea high-resolution, well-dated palynological-climatological investigation, the following conclusions can be drawn for the Bronze and Iron Ages: The Early Bronze through much of the Intermediate Bronze Age (ca. 3600–2200 BCE) was characterized by relatively wet climate conditions, with drier climate conditions emerging in ca. 2200/2100 BCE and through the beginning of the Middle Bronze I. The Middle Bronze II–III (ca. 1750–1550 BCE) shows once again wetter climate conditions. During the early phases of the Late Bronze Age, humid conditions continued. The driest conditions in the entire Bronze and Iron Age timespan were recorded towards the end of the Late Bronze Age (ca. 1250–1100 BCE), and this has been suggested as a contributing factor to the Late Bronze Age collapse [39, 40]. An increase in arboreal percentages was documented between ca. 1100 to 750 BCE, which covers most of the Iron I (ca. 1150–950) and the Iron IIA (ca. 950–780 BCE), showing humid conditions after the severe period of dryness. During the Iron IIB (ca. 780–680 BCE) and Iron IIC (ca. 680–586 BCE), the region experienced moderate climate conditions. A recent high-resolution, well-dated isotopic analysis of speleothems which examined the oxygen (δ^18^O) and carbon (δ^13^C) values within the Soreq Cave speleothems during the Bronze and Iron Ages revealed almost similar trends to those observed by the palynological studies [41]. This was also the case with the reconstruction of the Dead Sea lake levels [42, 43]. Slight dissimilarities may result due to different dating methods as well as variations in the sampling resolution and sensitivity of the paleoenvironmental proxies.

### 2.2 Vegetation

The high-altitude habitats of the Negev Highlands (700–1,000 m asl) are often characterized by tree and shrub cover, commonly found in areas with considerably higher precipitation, thus giving the vegetal landscape of this district a steppe-forest-like appearance [28, 44]. At these altitudes, the most common arboreal species is Mount Atlas pistachio (*Pistacia atlantica*). A regular companion of the *Pistacia*, but much less abundant, is the Two-seeded Buckthorn (*Rhamnus disperma*) and a less common companion is the Ramon almond (*Amygdalus ramonensis*). The open areas between the sparsely spaced trees are covered by largely diffuse shrub vegetation, primarily steppe-Irano-Turanian in character. The leading taxon in the shrub layer is white wormwood (*Artemisia sieberi*). At favorable habitats even more mesic shrubby species may be countered, such as leafless ephedra (*Ephedra foemina*; [28]). Most annuals germinate after the first significant rains, usually in the early winter, mature in the spring, and die before July.

At lower elevations, the vegetation becomes increasingly sparse and much more xeric. The flora displays a mosaic of Saharo-Arabian desert vegetation with tropical (Sudanian) elements. The wide wadi beds are dominated by white broom (*Retama raetam)*, chenopods, twisted acacia *(Acacia raddiana)*, and several tamarisk species (e.g., *Tamarix nilotica* and *T*. *aphylla)*. Large bushy bean caper (*Zygophyllum dumosum*) shrubs grow between the trees in the wadi beds and are also sporadically spread over the flanking slopes.

Until the Chalcolithic period, Junipers (*Juniperus phoenicea*) were also common in the Negev Highlands (late 6th and 5th millennia BCE; [45]). Archaeobotanical studies from the region indicate that during the late Pleistocene, a denser tree community existed in this area. The diminishing of the arboreal cover is attributed to human influence on the natural environment [45, 46]. The same phenomenon has recently been observed in the Middle Paleolithic Judean Desert [47], and in the Iron Age southern Arabah [26].

### 2.3 Archaeological sites

#### 2.3.1 Subsistence economy and chronology

Until recently, all periods represented in the Negev Highlands settlements were studied using traditional macroarchaeological methods. The scarcity of faunal and botanical remains resulted in an interpretational lacuna regarding subsistence economies during settlement peaks, as well as their exact duration (minute chronological differences could not be traced due to the lack of organic material suitable for radiocarbon dating). Hence all sites and features representing a given settlement peak have been considered as belonging to a single wave of activity. In other words, both the period-by-period interpretation and the overall, long-term, socio-economic reconstruction of human activity in the Negev Highlands have been based on partial data. In order to overcome this problem, we collected materials for our palynological investigation only from sites that were recently dated at a higher resolution by ^14^C and/or OSL dating [8, 17, 48, 49]. The four palynologically studied sites are presented below with their updated chronology. Note that recent investigation indicates that throughout the Bronze and Iron Ages, the Negev people must have had access to round, dug and built water cisterns that dot the landscape of the region [50].

#### 2.3.2 Nahal Boqer 66

**Fig 4:**
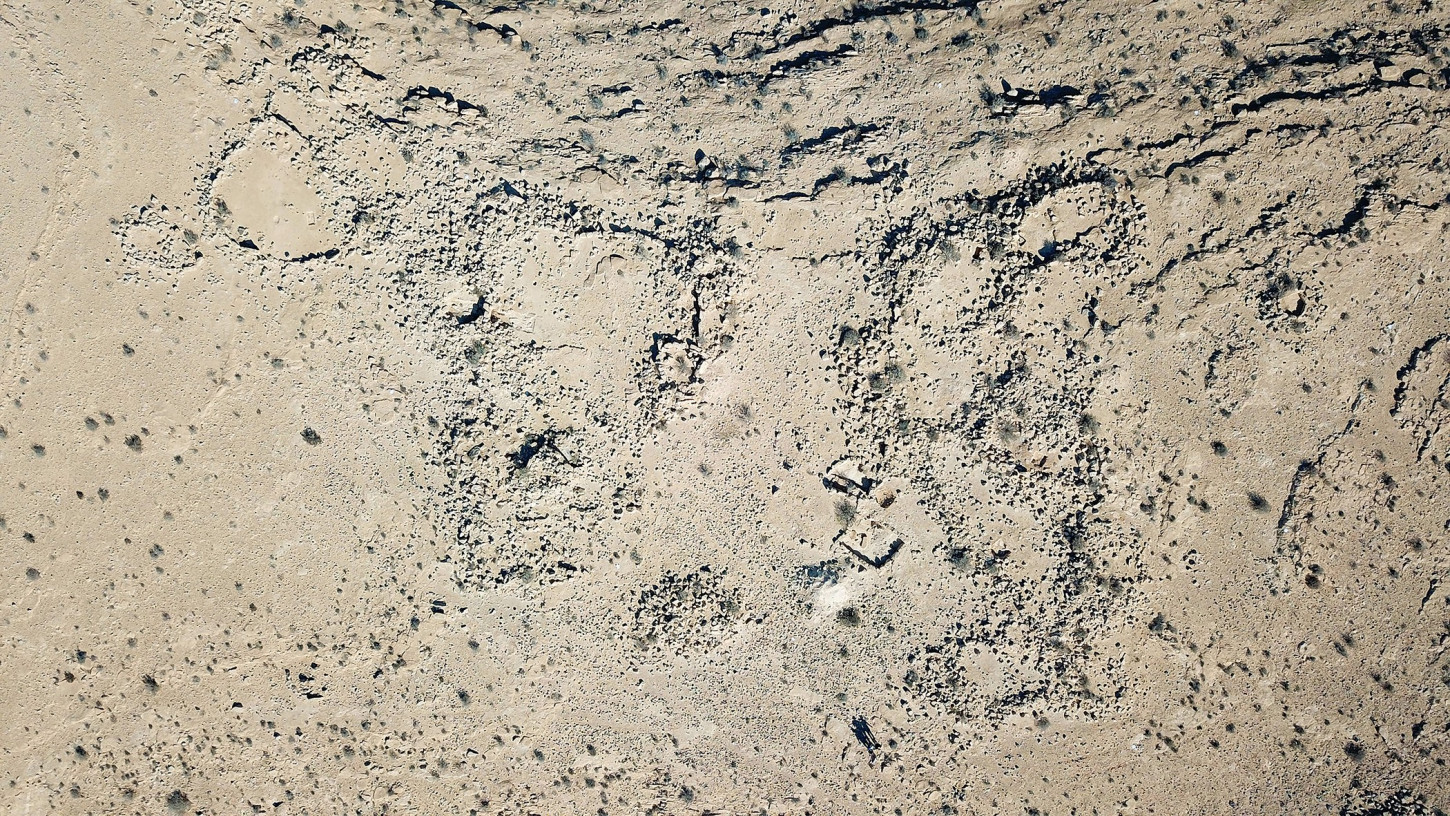
Nahal Boqer 66 aerial photo (taken by O. Ze’evi-Berger, courtesy of I. Finkelstein).

Nahal Boqer 66 (New Israeli Grid [NIG]: 1799/5354, ca. 520. m asl), is a small site (0.2 hectares), ca. 4 km north of modern Sede Boqer (Figs 1 and 4). The site is composed of two stone-built complexes typical of the Negev Highlands [6: p. 37–49; 21], each with oval or rectangular rooms (ca. 3–5 m in diameter) attached to large courtyards (8–14 m in diameter). Most walls are preserved only to 1–2 courses (ca. 50–60 cm) and are made of limestone blocks from the immediate vicinity. The site was excavated in the 1970s by Cohen [13: p. 60]. Lithic evidence suggests Pre-Pottery Neolithic B activity [51], while the structures were dated by ceramic typology to later periods. The northern complex is dominated by Intermediate Bronze Age sherds. At the southern complex a mix of Early Bronze Age and Intermediate Bronze Age sherds was exposed [13: p. 60]. No faunal or botanical remains were found during Cohen’s excavations. A recent set of ^14^C determinations from the site provided evidence for activity from the Early Bronze Age IB through the first half of the Intermediate Bronze Age (ca. 3200–2200 BCE), most probably in the form of discontinuous, short-term presence [8]. The microarchaeological investigation conducted by Dunseth et al. [19] suggests that the site was occupied by a desert nomadic pastoralist community.

#### 2.3.3 Ein Ziq

**Fig 5:**
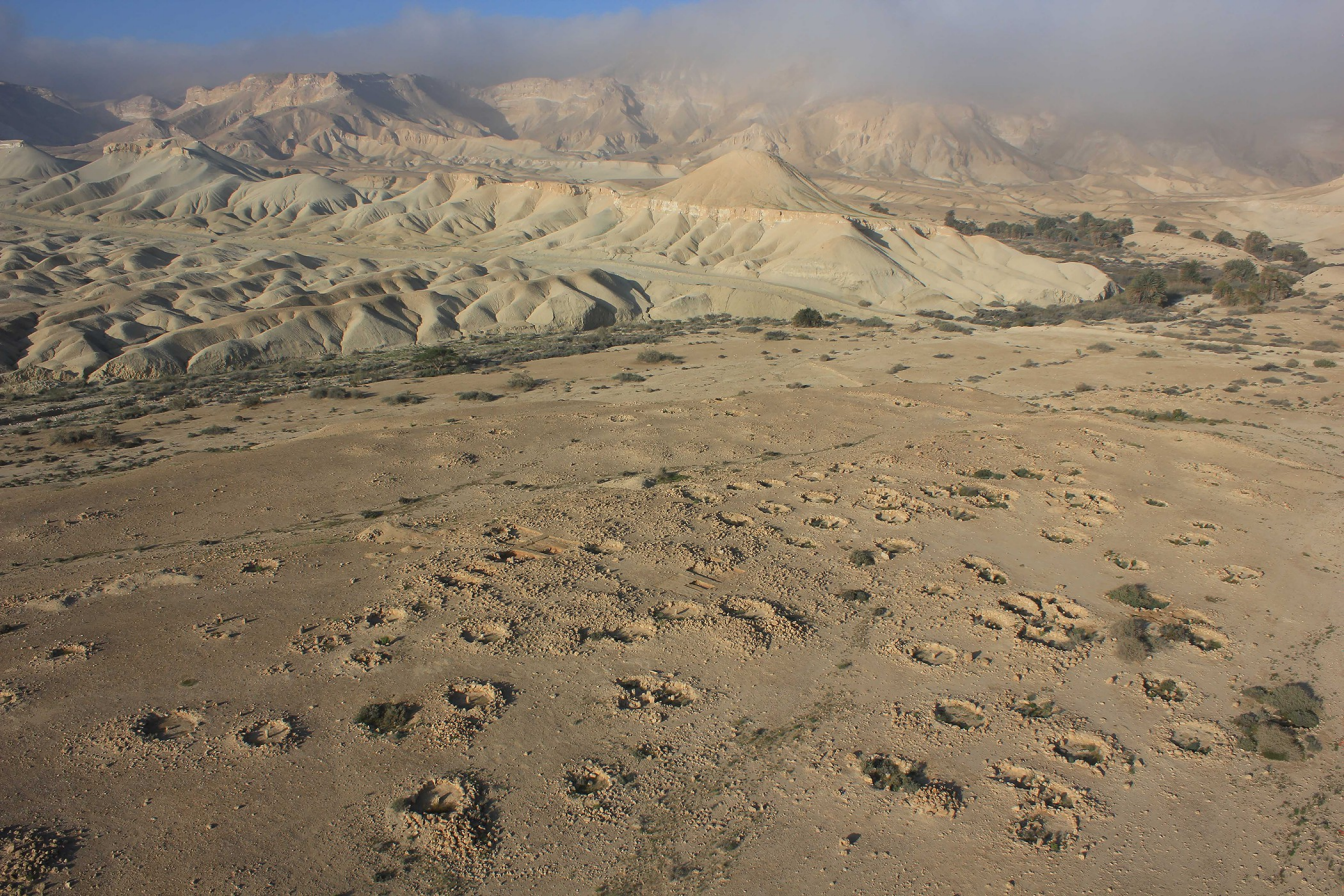
Ein Ziq. The excavated areas investigated in this study by the renewed expedition can be seen in the center of the image. Photo courtesy of I. Finkelstein.

Ein Ziq (NIG: 1861/5238, ca. 320 m asl) is the largest Intermediate Bronze Age site in the Negev Highlands, spreading over 2 ha of a pair of alluvial terraces above the dry wadi bed of Nahal Ziq (Figs 1 and 5). Two natural springs—Ein Ziq and Ein Shaviv—are in the immediate vicinity, ca. 1 km southwest of the site. The site was extensively excavated (110 rooms/structures out of over 200 visible on the surface) in the early 1980s by Cohen [13: p. 137–188]. Structures at the site (ca. 2–4 m in diameter) are all stone-built and preserved in some areas up to 1 m. Ceramics found in excavation showed one main period of activity, during the Intermediate Bronze Age, with some later reuse of structures in the Nabatean and Early Islamic periods [52]. Radiocarbon dates from the Intermediate Bronze Age structures in both Cohen’s excavation and a recent excavation conducted by Dunseth showed an almost exclusively short range of activity limited to the first half of the Intermediate Bronze Age (ca. 2,500–2,200 BCE, [8]). Wood remains recovered in Cohen’s excavation pointed to the presence of natural woody desert plants only, with no evidence of fruit tree horticulture [53]. Moreover, the microarchaeological investigation performed by Dunseth et al. [19] showed no association with food production (herding, agriculture) or metallurgical activities; according to the authors, the site should be understood as a trading hub.

#### 2.3.4 Mashabe Sade

**Fig 6:**
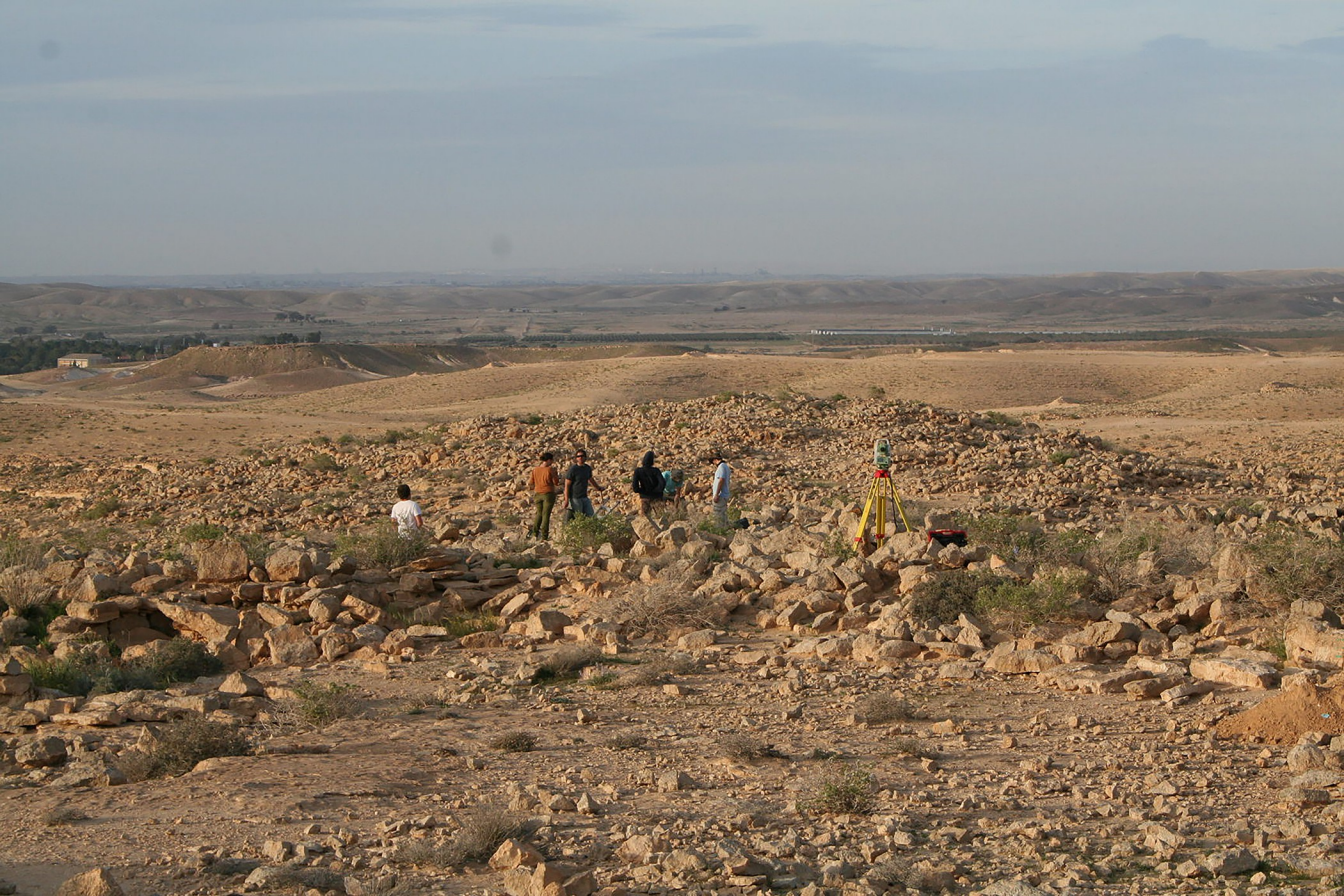
Mashabe Sade during the 2013 field excavation. The photo was taken by I. Finkelstein.

Mashabe Sade (NIG: 1798/5443; ca. 390 m asl) covers an area of 1.2 ha, with over 200 rounded, stone-made structures visible on the surface (Figs 1 and 6), making it one of the largest Intermediate Bronze Age settlements in the Negev Highlands [18, 49]. Salvage excavations were carried out in 1984 [13: p. 117–129]. Cohen cleared 21 structures of local fieldstones built directly on bedrock. Each room had one or two central drum-built pillars supporting stone roofing slabs; walls were preserved to an approximate height of 0.6–1.2m. Intermediate Bronze Age sherds and vessels were found in most loci, including complete bowls, storage jars and amphorae. The finds also included a copper dagger, a fragment of an ingot and grinding stones. Faunal remains were few and inconclusive [54]. No open enclosures or central courtyards were identified. Two short, limited-in-scope excavation seasons (January and March 2013) were conducted by Dunseth et al. [18]. Based on OSL dating, the sediments which accumulated on the site’s structures immediately postdate the Intermediate Bronze Age (ca. 1950–1650 BCE; [49]). The microarchaeological evidence points to the total absence of evidence of food production—herding and/or agriculture—as well as metallurgical activities [18]. According to the excavators, Mashabe Sade should also be understood as a trading hub.

#### 2.3.5 Haroa

**Fig 7:**
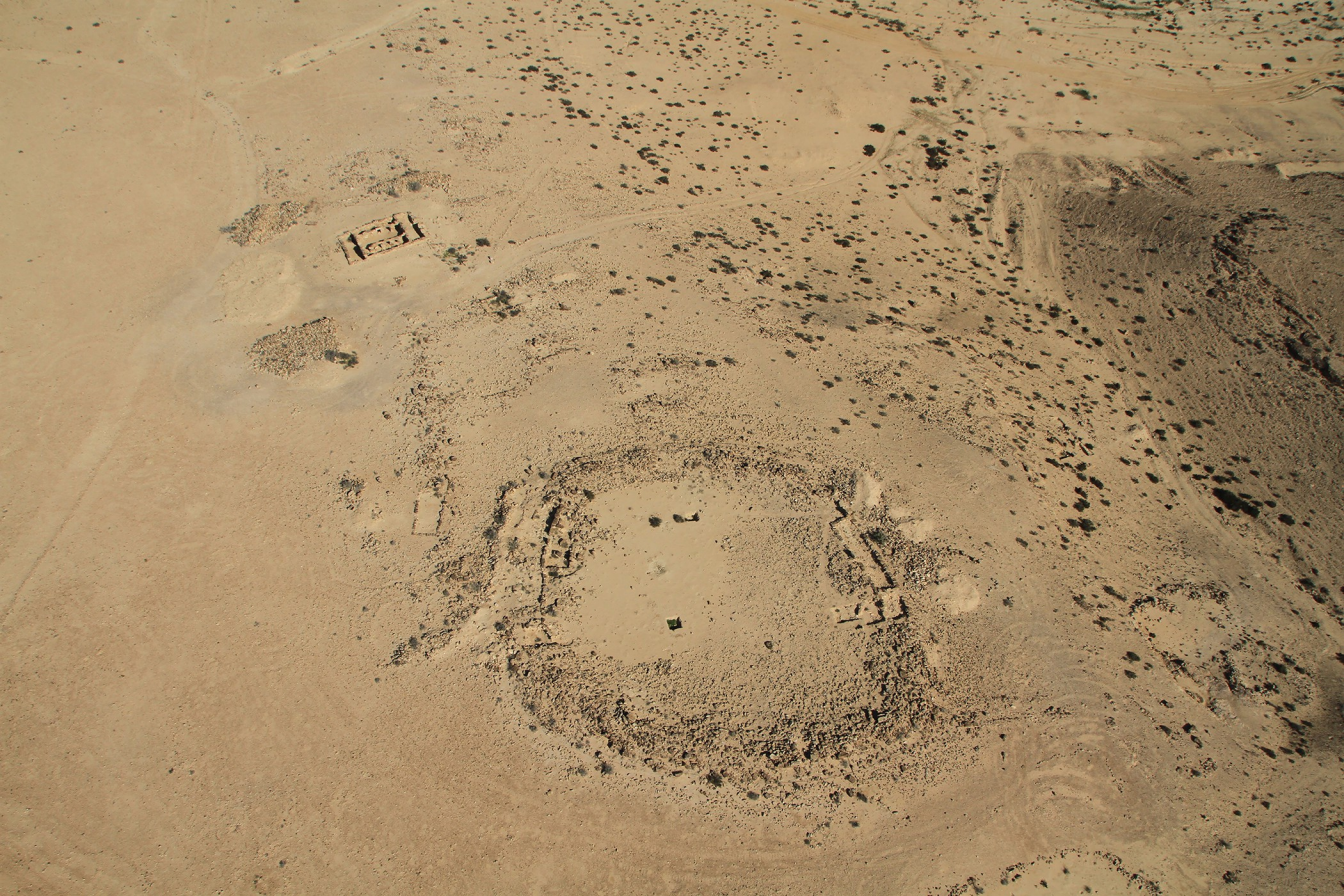
Aerial photo of Haroa site. At the bottom right, Haroa rock shelter can be seen. Photo courtesy of I. Finkelstein.

Haroa (NIG: 1797/4294; 550 m asl; Fig 1) is located ~7 km northeast of Sede Boqer. The site is built on a low hill overlooking a broad valley with deposits of alluvial sediments (Fig 7). The site was excavated in the 1960s by Cohen [55: p. 176–185]. It includes an oval compound with a belt of broad rooms surrounding a large courtyard, a group of pillared houses and other elements that have been interpreted as a threshing floor and field terraces. Cohen excavated several broad rooms in the oval structure (described as “casemates”) and one pillared house. He identified the oval compound as an Israelite fortress that was built in the days of King Solomon (traditionally 970–931 BCE) in order to control the southern trade routes and protect the borders of the kingdom and suggested that it was destroyed during the military campaign to Canaan undertaken by Pharaoh Sheshonq I in the late 10th century BCE. Shahack-Gross and Finkelstein carried out a geoarchaeological and dating project at the site in 2005–2006 [15, 17]. Their radiocarbon results placed activity at the site in the 9th century BCE, with a possibility that it was founded in the second half of the 10th century BCE [48]. The results of the microarchaeological investigation suggest that the inhabitants subsisted on animal husbandry, without practicing cereal cultivation [15].

## 3. Materials and method

### 3.1 Field sampling

In order to trace subsistence practices in the Negev Highlands sites, samples for palynological analyses were collected during field excavations from the four sites described above, which represent the waves of settlement in the region during the Bronze and Iron Ages. Samples were retrieved from uncontaminated, secure archaeological contexts by using sterile equipment; for each site, our attempt was to collect samples from surface activities of inner architectural structures as well as from contexts outside the buildings such as courtyards. Recent and sub-recent sediment samples were taken for control purposes in order to reveal the modern pollen rain. The control samples were collected from various contexts (e.g., surface sediments, modern rock shelters, and modern dung pellets). A total of 54 samples were analyzed. All necessary permits from the Israel Antiquity Authority were obtained for the collection of the pollen samples analyzed in this study, which complied with all relevant regulations.

Nine samples were retrieved from Nahal Boqer 66 during the 2016 excavation (Fig 4). Three samples were collected from light pale brown sediments which accumulated on the bedrock—the structures’ floors (samples P1-P3), and five samples were taken from grey sediments, shown to be often related to dung and/or ash deposits ([e.g., 17]; samples P4-P8). A recent livestock dung pellet found near the site was used as a control sample (P9). Another dung pellet was collected from the nearby wadi of Nahal Noked, also used for control purposes (sample no. P10). According to the study conducted by Dunseth et al. [19], samples P4-P6 were recovered from Early Bronze Age III and early Intermediate Bronze Age contexts, while samples P7 and P8 originated from Early Bronze Age IB-II contexts.

Ten samples were taken from Ein Ziq during the 2014–2015 excavations (Fig 5). Two samples were recovered from the courtyard of an Intermediate Bronze Age structure (P11 and P12) and six other samples (P13-P18) were retrieved from an activity surface of a structure. Another sample (P19) was collected from the accumulation layer of charcoals and sediments within a courtyard, which may represent an ash deposit. For control purposes, soft yellow sub-recent dust sediment was taken below the desert pavement (P20).

At the Intermediate Bronze Age site of Mashabe Sade pollen was extracted from eleven sediment samples (P21-P31) during the two short winter excavations in 2013 (Fig 6). The samples were retrieved from various contexts: samples P21-P25 were recovered from the floors of the excavated structures (that is, sediments that accumulated on top of the bedrock). Samples P26-P27 were taken from open courtyards. Four control samples were collected: P28 from the nearby rock shelter of Umm Urgan, where 1.2 m fine grey sediments were accumulated. The sample was taken at a depth of 40 cm. Two sub-recent samples were collected from the site at depths of -5 cm and -10 below the surface (P29, P30, respectively), and one surface sediment was also taken (P31).

At Haroa site one sample was analyzed from the central open courtyard (P32) and two samples were taken from surface activities (P33-P34, collected during the 2005 season excavation). According to Boaretto et al. [48] and Shahack-Gross et al. [17], these samples derive from Iron Age II contexts. Sample P35 was taken from the surface sediment of the site for control purposes. In March 2015 a small-scale excavation was conducted by the authors at an open rock shelter located on the eastern side of the oval site (Fig 7). Fifteen samples were extracted along a 1.4 m sediment sequence at a 10 cm interval (P36-P50). For comparison, four samples were collected from Nahal Mesora rock shelter (NIG: 1712/5373) at a 20 cm interval from one sample to another (P51-P54).

### 3.2 Palynological analyses

Pollen extraction followed a physical-chemical preparation procedure common to Levantine desert sediments [56]: One *Lycopodium* spore tablet was added to each sample in order to calculate pollen concentrations [57]. Samples were then immersed in HCl to remove the calcium carbonates, and a density separation was carried out using a ZnBr_2_ solution (with a specific gravity of 1.95) in order to float the organic material, together with three minutes of sonication. After sieving (150 μm mesh screen) and short acetolysis, the unstained residues were homogenized and mounted onto microscopic slides using glycerin. Pollen grains were identified under a light microscope, with magnifications of 200X, 400X and 1,000X (oil immersion), to the most detailed systematic level. For each sample, the entire extracted residue was counted. For pollen identification, a comparative reference collection of the Israeli pollen flora of Tel Aviv University (Steinhardt Museum of Natural History) was used, in addition to pollen atlases [58–62].

## 4. Results

### 4.1 Pollen preservation

Only 31 out of the 54 analyzed samples contained well-preserved pollen and were in satisfactory concentration to allow identification and interpretation of the pollen spectra. The results of these samples are summarized in Tables 1–4. Higher pollen concentrations were calculated for samples that were related to dung (samples from rock shelters, sediments identified as degraded dung and pellets), in comparison to pollen that was recovered from activity surfaces (buildings and open courtyards). Pollen grains are generally resistant to degradation by digestive enzymes and therefore can survive in dung remains. The different well-preserved pollen spectra that were identified are presented and explained below, for each of the four sites. When relevant, we relate to various aspects such as seasonality, taxa variety, and long-distance vs. short-distance pollen transportation (wind vs. insect-pollinated taxa, respectively).

**Table 1:** Pollen results from Nahal Boqer 66 (NB), dated to the Early and Intermediate Bronze Ages, together with two recent dung pellet control samples from Nahal Boqer and Nahal Noked (NN); raw data (absolute numbers) and percentages are presented. EB = Early Bronze. IB = Intermediate Bronze.

Field ID	NB		NB		NB		NB		NB		NB		NN	
	L8-2		L.8-1		L. 8–3		L.2-8		L. 2–7		recent		recent	
						
Lab ID	#P4		#P5		#P6		#P7		#P8		#P9		#P10	
Archaeological context Taxa	EBIII/ early IB degraded dung		EBIII/ early IB degraded dung		EBIII/ early IB degraded dung		EBIB-EBII degraded dung		EBIB-EBII degraded dung		modern dung pellet		modern dung pellet	
*Pinus halepensis* (Aleppo pine)	1	0.1		0.0		0.0		0.0	4	1.8	2	0.9		
*Olea europaea* (olive)		0.0		0.0		0.0		0.0		0.0		0.0	4	0.6
Cupressus/Juniperus (cypress/juniper)		0.0		0.0		0.0		0.0	1	0.5		0.0		
*Acacia* (acacia)		0.0		0.0		0.0		0.0		0.0	2	0.9	16	2.5
Asteraceae Asteroideae type (aster-like)	1161	98.1	391	70.3	195	94.7	8	2.8	2	0.9	6	2.6	16	2.5
Asteraceae Cichorioideae type (dandelion-like)		0.0		0.0		0.0	1	0.3		0.0	3	1.3	6	0.9
*Artemisia* (sagebrush)	3	0.3		0.0	3	1.5		0.0		0.0		0.0	6	0.9
*Centaurea cyanoides* (Syrian cornflower-thistle)		0.0		0.0		0.0		0.0		0.0		0.0	1	0.2
Chenopodiaceae (goosefoot family)	8	0.7	145	26.1	4	1.9	239	83.6	192	88.5	171	74.6	390	60.8
*Noaea* type (thorny saltwort)	4	0.3	5	0.9	1	0.5	2	0.7	3	1.4	5	2.2	111	17.2
Poaceae (grasses)		0.0		0.0		0.0		0.0	1	0.5		0.0	7	1.1
Caryophyllaceae (pink family)		0.0		0.0		0.0		0.0		0.0		0.0	10	1.6
Geraniaceae (cranesbill family)		0.0		0.0		0.0		0.0		0.0		0.0	1	0.2
Convolvulaceae (bindweeds)		0.0		0.0		0.0		0.0		0.0		0.0	11	0.7
Liliaceae (lilies)		0.0		0.0		0.0		0.0	1	0.5		0.0		0.0
Apiaceae (parsley family)		0.0		0.0		0.0	4	1.4		0.0		0.0	3	0.5
*Ephedra fragilis* type (joint pine)		0.0		0.0		0.0		0.0		0.0	2	0.9	2	0.3
Brassicaceae (cabbage family)	2	0.2	7	1.3		0.0	27	9.4	3	1.4	15	6.6	14	2.2
Fabaceae (legumes)		0.0	1	0.2		0.0		0.0		0.0		0.0	1	0.2
Lamiaceae (mint family)		0.0		0.0		0.0		0.0		0.0		0.0	1	0.2
Malvaceae (mallows)		0.0		0.0		0.0		0.0		0.0	8	3.5		
Polygonaceae (buckwheat)		0.0		0.0		0.0		0.0		0.0	2	0.9		
Rubiaceae (madder family)													2	0.3
Total pollen	1,184	100	556	100	206	100	286	100	217	100	229	100	642	100
Undetectable	5		7		3		5		10		13		38	
Spores							63		68				679	
Total palynomorphs	1,189		563		209		354		295		241			
*Lycopodium*	91		92		188		407		252		49		28	
Concentrations (gram/sediment)	40,213		18,816		3,424		2,679		3,380		129,859		112,047	
Weight (gram)	3.0		3.0		3.0		3.0		3.2		0.4		2.0	
Clumps of Chenopodiaceae	12		6				10		4		5		7	

**Table 2:** Pollen results from Ein Ziq (EZ), dated to the Intermediate Bronze Age; raw data (absolute numbers) and percentages are presented. IB = Intermediate Bronze.

Field ID	EZ 324		EZ 40		EZ 329		EZ 39		EZ control	
Lab ID	#P11		#P12		#P18		#P19		#P20	
Archaeological context Taxa	IB courtyard square		IB courtyard square		IB floor structure		IB waste accumulation		sub-recent sediments below desert pavement	
*Pinus halepensis* (Aleppo pine)		0.0	4	1.8		0.0	2	0.6		0.0
*Tamarix* (tamarisk)		0.0		0.0	11	4.9		0.0		0.0
*Cupressus/Juniperus* (cypress/juniper)		0.0	8	3.7		0.0		0.0		0.0
Asteraceae Asteroideae type (aster-like)	6	18.2	11	5.0	57	25.2	6	1.9	1	0.9
Asteraceae Cichorioideae type (dandelion-like)		0.0	5	2.3	8	3.5	4	1.3		0.0
*Artemisia* (sagebrush)		0.0	25	11.4	9	4.0		0.0	17	16.0
*Xanthium* (cocklebur)		0.0	5	2.3		0.0		0.0		0.0
Chenopodiaceae (goosefoot family)	11	33.3	157	71.7	112	49.6	222	69.8	88	83.0
*Noaea* type (thorny saltwort)	14	42.4		0.0	1	0.4	6	1.9		0.0
Poaceae (grasses)	2	6.1		0.0	14	6.2	5	1.6		0.0
Poaceae (cereals)		0.0		0.0		0.0	6	1.9		0.0
Caryophyllaceae (pink family)		0.0	4	1.8		0.0		0.0		0.0
Geraniaceae (cranesbill family)		0.0		0.0	4	1.8	4	1.3		0.0
Apiaceae (parsley family)		0.0		0.0	5	2.2	2	0.6		0.0
Brassicaceae (cabbage family)		0.0		0.0	4	1.8	1	0.3		0.0
Urticaceae (nettle family)		0.0		0.0	1	0.4		0.0		0.0
Malvaceae (mallows)		0.0		0.0		0.0	3	0.9		0.0
Boraginaceae (borage family)		0.0		0.0		0.0	1	0.3		0.0
Rubiaceae (madder family)		0.0		0.0		0.0	1	0.3		0.0
Total pollen	33	100.0	219	100.0	226	100.0	318	100.0	106	100.0
Undetectable	3		7		34		55		71	
Spores	4		59		1		13			
Total palynomorphs	40		285		261		386		177	
*Lycopodium*	145		329		888		166		131	
Concentrations (gram/sediment)	1,593		2,582		754		6,139		4,162	
Weight (gram)	1.6		3		3.6		3.5		3	
Clumps of Chenopodiaceae	12		19		33		4		2	

**Table 3:** Pollen results from the central Mashabe Sade site dated to the Intermediate Bronze Age; raw data (absolute numbers) and percentages are presented.

Field ID	MS1		MS10		MS12		MS14		MS7	MS18		MS11		MS23		MS5		MS4 control		MS24 control
																	control		control			
Lab ID	#P21		#P22		#P23		#P24		#P25	#P26		#P27		#P28		#P29		#P30		#P31
Archaeological	floor		floor		floor		floor		5 cm above floor	open IB courtyard		open courtyard (dung mixed with ash)		rock-shelter Umm Urgan		sub recent sediments (5 cm below surface)		sub recent sediments (10 cm below surface)		surface sediments
context Taxon																						
*Quercus calliprinos* type (Kermes oak)	1	0.4		0.0		0.0		0.0		0		0		0		0		0.0		0.0		0
*Pinus halepensis* (Aleppo pine)		0.0	1	0.3		0.0		0.0		0.0	7	1.9	1	0.4		0.0	9	2.7	7	1.6	11	8.1
*Olea europaea* (olive)		0.0		0.0	2	0.4		0.0		0.0	2	0.5		0.0	1	0.6		0.0	2	0.5		0.0
*Tamarix* (tamarisk)	2	0.8		0.0		0.0		0.0		0.0		0.0		0.0		0.0		0.0		0.0		0.0
*Cupressus/Juniperus* (cypress/juniper)	1	0.4	2	0.6		0.0	4	2.3	7	3.1		0.0		0.0		0.0		0.0		0.0	11	8.1
*Ziziphus* (jujube)		0.0		0.0		0.0	3	1.7		0.0		0.0		0.0		0.0		0.0		0.0		0.0
Asteraceae Asteroideae type (aster-like)	4	1.7	4	1.1	1	0.2	4	2.3	10	4.5	14	3.8	1	0.4	9	5.0	11	3.3	14	3.2	25	18.5
Asteraceae Cichorioideae type (dandelion-like)	5	2.1	7	2.0	1	0.2		0.0	11	4.9	41	11.0	1	0.4	15	8.3	144	43.6	311	71.8	4	3.0
*Artemisia* (sagebrush)		0.0		0.0		0.0	13	7.5		0.0		0.0		0.0	81	45.0	3	0.9		0.0	10	7.4
*Echinops* (globe thistle)		0.0		0.0	1	0.2		0.0		0.0		0.0		0.0		0.0		0.0		0.0	7	5.2
*Centaurea cyanoides* (Syrian cornflower-thistle)	1	0.4		0.0	1	0.2	2	1.2		0.0		0.0		0.0		0.0		0.0		0.0		0.0
*Centaurea nigra* (black knapweed)		0.0		0.0		0.0	2	1.2	2	0.9	4	1.1		0.0		0.0		0.0		0.0		0.0
*Carthamus* (distaff thistle)	2	0.8		0.0	5	1.1	4	2.3		0.0	2	0.5		0.0		0.0		0.0		0.0	7	5.2
*Gundelia* (tumble thistle)		0.0		0.0		0.0	17	9.8		0.0		0.0		0.0		0.0		0.0		0.0		0.0
*Xanthium* (cocklebur)	3	1.3		0.0	2	0.4	25	14.5		0.0	7	1.9		0.0		0.0	2	0.6	2	0.5		0.0
*Atriplex* type (saltbush)	169	70.7	219	61.2	341	72.4	13	7.5	163	72.8	149	39.9	195	83.0	4	2.2	129	39.1	46	10.6	21	15.6
*Noaea* type (thorny saltwort)	5	2.1	25	7.0	4	0.8	2	1.2	11	4.9	11	2.9	14	6.0	8	4.4	2	0.6	4	0.9	3	2.2
Chenopodiaceae other (goosefoot family)	1	0.4	2		6	1.3	1	0.6	9		4	1.1	22				11	3.3	1	0.2	1	0.7
Poaceae (grasses)		0.0	2	0.6		0.0		0.0	3	1.3		0.0		0.0		0.0	1	0.3		0.0		0.0
*Helianthemum sancti-antonii* type (desert sunrose)	23	9.6	91	25.4	32	6.8	11	6.4		0.0	6	1.6		0.0		0.0	1	0.3		0.0		0.0
Caryophyllaceae (pink family)		0.0	4	1.1	7	1.5		0.0		0.0	64	17.2		0.0	27	15.0	1	0.3	27	6.2	4	3.0
Liliaceae (lilies)		0.0		0.0	1	0.2		0.0		0.0		0.0		0.0		0.0	3	0.9	3	0.7		0.0
Geraniaceae (cranesbill family)		0.0		0.0	1	0.2		0.0	4	1.8		0.0		0.0		0.0	2	0.6	6	1.4	1	0.7
*Erodium* (stork’s bill)		0.0		0.0		0.0	4	2.3		0.0		0.0		0.0		0.0		0.0		0.0		0.0
Dipsaceae (teasel family)		0.0		0.0		0.0		0.0		0.0	4	1.1		0.0		0.0	2	0.6		0.0		0.0
*Scabiosa* (scabious)		0.0		0.0	6	1.3	4	2.3		0.0	15	4.0		0.0		0.0	2	0.6	4	0.9	1	0.7
*Convolvulus* (bindweed)					2	0.4																
Convolvulaceae (bindweeds)		0.0		0.0	21	4.5		0.0		0.0	11	2.9		0.0		0.0		0.0		0.0		0.0
Apiaceae (parsley family)		0.0		0.0	1	0.2		0.0		0.0	12	3.2		0.0	2	1.1		0.0		0.0		0.0
*Bunium* type					5	1.1					13	3.5										
*Turgenia* type (false carrot)					2	0.4						0.0										
*Ephedra fragilis* type (joint pine)	2	0.8		0.0		0.0		0.0	4	1.8	2	0.5		0.0		0.0	3	0.9	2	0.5	2	1.5
Brassicaceae (cabbage family)	19	7.9	1	0.3	4	0.8		0.0		0.0	4	1.1		0.0	16	8.9	1	0.3	2	0.5	26	19.3
Ranunculaceae (buttercup family)		0.0		0.0		0.0		0.0		0.0		0.0		0.0	5	2.8		0.0		0.0		0.0
Fabaceae (legumes)		0.0		0.0	4	0.8	64	37.0		0.0		0.0		0.0		0.0	1	0.3		0.0		0.0
*Vicia* type (vetches)					7																	
*Limonium* (sea lavender)					5																	
Urticaceae (nettle family)		0.0		0.0		0.0		0.0		0.0		0.0		0.0	11	6.1		0.0		0.0		0.0
Lamiaceae (mint family)		0.0		0.0		0.0		0.0		0.0		0.0		0.0		0.0	1	0.3		0.0		0.0
Plantaginaceae (plantain family)	1	0.4		0.0		0.0		0.0		0.0	1	0.3		0.0		0.0	1	0.3	2	0.5	1	0.7
Malvaceae (mallows)		0.0		0.0		0.0		0.0		0.0		0.0	1	0.4		0.0		0.0		0.0		0.0
Boraginaceae (borage family)		0.0		0.0		0.0		0.0		0.0		0.0		0.0	1	0.6		0.0		0.0		0.0
*Crocus* (croci)					2																	
Rubiaceae (madder family)		0.0		0.0	3	0.6		0.0		0.0		0.0		0.0		0.0		0.0		0.0		0.0
Valerianaceae (valerian family)					2																	
*Daphn*e/Thymelaea		0.0		0	2	0		0		0		0		0		0		0		0		0
Total pollen	239	100.0	358	100.0	471	100.0	173	100.0	224	100	373	100	235	100	180	100	330	100.0	433	100.0	135	100
Undetectable	9		10				31		99				1		27				11		74	
Spores	4		4		1		19		13		4		191		2		10		20		83	
Terfeziaceae spore type (desert truffle)											4						3					
Total palynomorphs	252		372		472		223		336		377		427		209		340		464		292	
*Lycopodium*	195		56		97		1198		591		242		211		251		181		339		53	
Concentrations (gram/sediment)	3,852		14,972		12,491		441		1,281		3,788		4453		2137		4234		4,216		11,840	
Weight (gram)	3.1		4.1		3.6		3.9		4.1		3.8		4.2		3.6		4.1		3		4.3	
Clumps of Chenopodiaceae	23		62		57		12		43		23		52		1		7		8		7	

**Table 4:** Pollen results from the Iron Age II Haroa site; raw data (absolute numbers) and percentages are presented.

	RAR 58		RAR 84		Rock shelter*		Rock shelter*		Rock shelter*		Rock shelter*		Rock shelter*		Rock shelter*	
					surface		-10 cm		-20 cm		-30 cm		-40 cm		-50 cm	
	central courtyard		control surface		Modern dung		Sub-modern dung		Sub-modern dung		Sub-modern dung		Sub-modern dung		Sub-modern dung	
	#P32		#P35		#P36		#P37		#P38		#P39		#P40		#P41	
							
*Pinus* (pine)		0.0	2	0.5	25	10.3	13	4.6		0.0	3	1.0	2	0.6	2	0.5
*Olea europaea* (olive)		0.0	1	0.2	2	0.8	3	1.1		0.0	2	0.6		0.0		0.0
*Tamarix* (tamarisk)		0.0		0.0		0.0		0.0		0.0		0.0		0.0		0.0
*Cupressus/Juniperus* (cypress/juniper)		0.0	1	0.2		0.0		0.0		0.0		0.0		0.0		0.0
*Acacia* (acacia)		0.0		0.0		0.0		0.0	2	0.7		0.0	5	1.5		0.0
Asteraceae Asteroideae type (aster-like)	24	5.9	69	16.7	35	14.4	12	4.3	42	14.6	29	9.4	2	0.6	71	17.0
Asteraceae Cichorioideae type (dandelion-like)	2	0.5	68	16.4	10	4.4	6	2.1	5	1.7	28	9.1	31	9.6	22	5.3
*Artemisia* (sagebrush)	1	0.2	15	3.6	11	4.5	8	2.9		0.0	6	1.9	9	2.8	36	8.6
*Centaurea* (knapweed)	7	1.7		0.0		0.0		0.0		0.0		0.0		0.0		0.0
*Gundelia* (tumble thistle)	2	0.5	1	0.2		0.0		0.0		0.0		0.0		0.0		0.0
*Xanthium* (cocklebur)	31	7.6	1	0.2	3	1.2		0.0		0.0	4	1.3	1	0.3	5	1.2
*Atriplex* (saltbush)	61	15.0	111	26.8	43	17.7	155	55.3	188	65.5	77	25.0	143	44.1	111	26.6
*Noaea* (thorny saltwort)	259	63.8	86	20.8	3	1.2	1	0.4	41	14.3	32	10.4	85	26.2	114	27.3
Chenopodiaceae other (goosefoot family)	2	0.5	20	4.8	5	2.1	5		3	1.0	55	17.9	11	3.4	20	4.8
Poaceae (grasses)		0.0		0.0		0.0		0.0		0.0		0.0	4	1.2		0.0
Poaceae (cereals)		0.0		0.0		0.0		0.0		0.0		0.0		0.0	4	1.0
*Helianthemum* (sunrose)		0.0		0.0		0.0		0.0		0.0	1	0.3	2	0.6	8	1.9
Caryophyllaceae (pink family)	5	1.2	12	2.9	2	0.8		0.0	3	1.0	22	7.1	1	0.3	2	0.5
Liliaceae (lilies)		0.0	1	0.2		0.0	2	0.7		0.0	1	0.3	2	0.6	3	0.7
Geraniaceae (cranesbill family)	1	0.2	14	3.4	15	6.2		0.0		0.0	1	0.3		0.0		0.0
Apiaceae (parsley family)	1	0.2		0.0		0.0		0.0		0.0		0.0		0.0		0.0
*Ephedra fragilis* type (joint pine)		0.0		0.0	2	0.8		0.0	2	0.7	5	1.6		0.0		0.0
Brassicaceae (cabbage family)	2	0.5	9	2.2	46	18.9	37	13.2		0.0	25	8.1	17	5.2	19	4.5
Fabaceae (legumes)		0.0		0.0	2	0.8		0.0		0.0		0.0		0.0		0.0
*Vicia* (vetches)												0.0			1	0.2
Urticaceae (nettle family)	1	0.2		0.0		0.0		0.0		0.0		0.0	1	0.3		0.0
Dipsaceae (teasel family)		0.0	1	0.2		0.0		0.0		0.0		0.0		0.0		0.0
*Scabiosa* (scabious)	3	0.7		0.0		0.0		0.0		0.0	1	0.3	1	0.3		0.0
Malvaceae (mallows)		0.0		0.0	39	16.0	36	12.9	1	0.3	14	4.5	6	1.9		0.0
*Crocus* (croci)							2	0.7		0.0	1	0.3		0.0		0.0
*Daphne*/Thymelaea		0.0	2	0.5		0.0		0.0		0.0	1	0.3	1	0.3		0.0
Cyperaceae (sedges)	4	1.0														
Total pollen	406	100.0	414	100.0	243	100.0	280	100.0	287	100.0	308	100.0	324	100.0	418	100.0
Undetectable	7		8		44		1		11		19		9		29	
Spores	2		11		399		123		97		60		15		44	
Total palynomorphs	415		433		686		404		395		387		348		491	
*Lycopodium*	323		212		509		15		39		66		44		36	
Concentrations (gram/sediment)	3,830		4,840		4,151		88,890		25,999		14,259		19,234		36,011	
Weight (gram)	3.1		3.9		3		2.8		3.6		3.8		3.8		3.5	
Clumps of Chenopodiaceae	29		2		5		12		11		20		35		13	

*Pollen diagram of Haroa rock shelter is available at S1 Fig.

### 4.2 Characterization and interpretation of the palynological assemblages

#### 4.2.1 Nahal Boqer

The three samples that were retrieved from the light pale brown sediments suggested as belonging to surface activity were pollen barren (P1-P3). All the samples collected from the grey sediments contained well-preserved pollen grains (samples nos. P4-P8; Table 1). This observation, together with the microscopic identification of dung spherulites [19] confirms that these grey sediments are related to degraded dung deposits rather than to ash (pollen could not have been preserved in an ash context due to the high temperatures). Indeed, the resemblance of the pollen spectra of the recent dung pellets (samples nos. P9-P10; Table 1) to the archaeological samples, and more specifically to Samples P7 and P8, supports the notion that the pollen originated from dung deposits. Samples P4-P6 that derived from Early Bronze Age III and early Intermediate Bronze Age contexts are dominated by Asteraceae Asteroideae pollen type (aster-like) and are characterized by medium pollen concentrations (40,213–18,816 g/sediment). Samples P7 and P8 that derive from Early Bronze Age IB-II contexts are dominated by Chenopodiaceae (goosefoot family) pollen and are typified by low pollen concentrations (3,380–2,679 pollen/g/sediment). The overlap in the blossoming months of the two dominant wild taxa covers the spring season: the aster pollen type growing around the study area blooms between February and May, whereas members of the goosefoot family bloom between March to November. The total absence of autumn bloomer plants such as acacia and wormwood from the Early Bronze IB-II samples (P7 and P8) seems to suggest that all palynological spectra (P4-P8) originated from spring blooming plants. Herding activity was therefore limited to the spring. The presence of pollen grains in clumps in almost all samples most probably indicates that the entire inflorescence had been eaten (pollen grains are attached to one another in the flower source). The slightly higher species diversity and the relatively high pollen concentrations, which were found in the modern dung (129,859 and 112,047 pollen/g/sediment; Samples P9 and P10, respectively), most likely relate to a better state of pollen grain preservation and their occurrence in pellet versus sediment deposits of degraded livestock dung. The modern samples, unlike the archaeological ones, included pollen of autumn blooming plants, in addition to spring and summer blooming plants. Indeed, today herding is carried out in the region year-round.

#### 4.2.2 Ein Ziq

Only five of the ten analyzed samples contained pollen (Table 2). The successful samples (P11, P12, P18-P20) are composed mostly of desert components. The presence of 1.9% of cereal pollen in sample P19 is interesting since this is almost the only sample analyzed in this study that yielded evidence of cereal. Dunseth et al. [19] also reported similar findings—dendritic phytoliths were recovered from a hearth context. According to the authors, these are the remains of grass inflorescences used as tinder, as hearths were not fueled by dung. The other pollen (this study) and phytolith assemblages [19, 63] which were analyzed from Ein Ziq show no clear signs of domestic cereals. Microscopic examination of all samples studied from Ein Ziq revealed no evidence of dung spherulites [19]. The components of the palynological spectra together with their relatively low/medium concentrations (754–6,139 pollen/g/sediment) corroborate this observation. Pollen concentrations are lower than those calculated for Nahal Boqer 66 and identified as dung. In addition, the Ein Ziq palynological assemblages of the courtyard and the ash deposit also include pollen of taxa which are characterized by long-distance wind transportation (Aleppo pine and cypress/juniper). The low pollen concentration together with the airborne pollen most probably points to a natural deposition of the ancient pollen rain (mainly by wind), rather than pollen accumulation due to animal and/or anthropogenic activity. The sub-modern sample is characterized by low taxa diversity, probably as a result of unfavorable preservation conditions.

#### 4.2.3 Mashabe Sade

**Fig 8:**
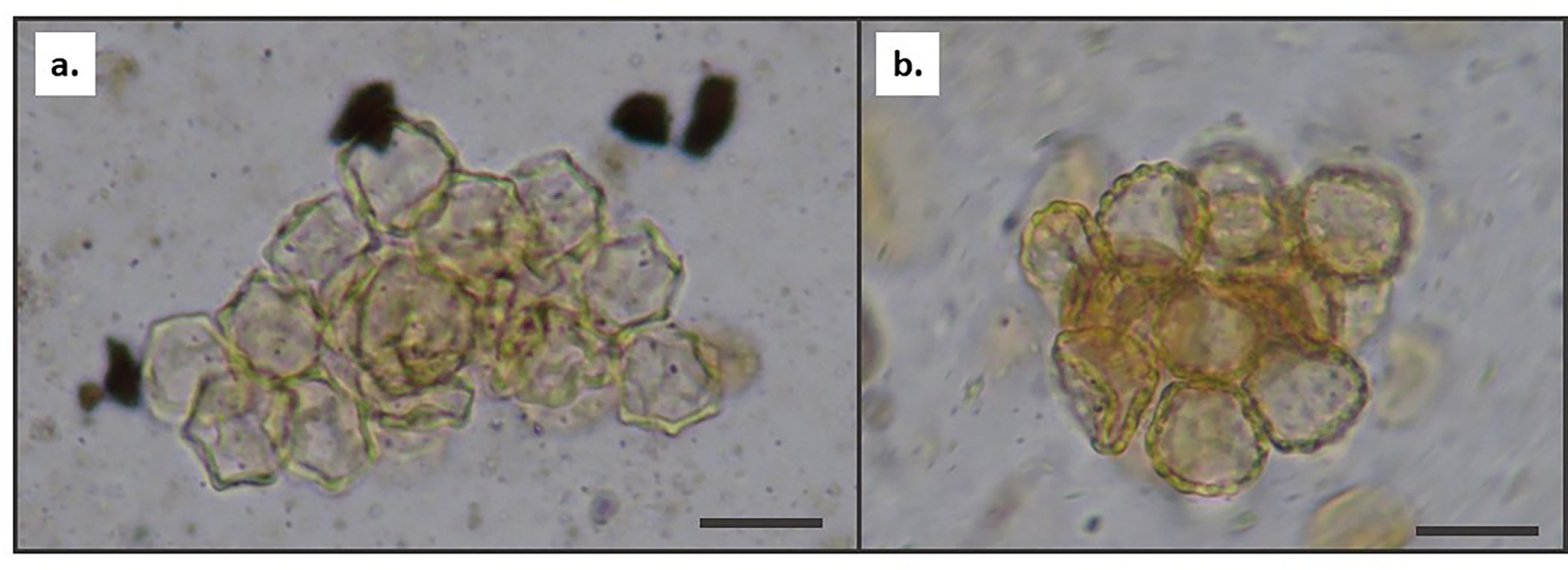
Pollen grains of Chenopodiaceae in a clump. (a) Noaea pollen type. (b). Atriplex pollen type. Clumps were extracted from Mashabe Sade site (sample P23). Photo was taken under a light microscope by D. Langgut. Bar represents 20 microns.

All eleven analyzed samples from the central Intermediate Bronze site of Mashabe Sade yielded pollen. Most archaeological samples (P21-P27) are dominated by wind-pollinated taxa of chenopods. The presence (though in very low ratios) of Mediterranean air-borne trees such as Aleppo pine, olive and cypress/juniper also points to pollen accumulated over a long distance. The high frequencies of chenopods in all of the samples extracted from structures together with their appearance in clumps (Table 3 and Fig 8) may also suggest that Chenopodiaceae (goosefoot) branches were used for roofing. This suggestion is based on archaeobotanical evidence from the 23,000 cal. years BP fisher campsite of Ohalo II. The charcoal assemblage of the brush huts at the site included charred wood remains of Chenopodiaceae [64: Fig 6] as well as pollen grains of Chenopodiaceae, some of them appearing in clumps [65]. Sample P23 which was recovered from the surface activity of one of the structures features a unique pollen assemblage in comparison to both the archaeological and the control samples (P28-P31). It is characterized by relatively high pollen concentrations (12,491 pollen/g/sediment) and the highest taxa diversity identified in this study; the majority are insect-pollinated plants and therefore most probably represent the nearby surrounding vegetation of the site. The identification of edible plants points to gathering possibilities that were available to the inhabitants, among them: *Vicia* (vetches), *Crocus* (croci), *Limonium* (Sea Lavender), *Gundelia* (tumble thistle), *Erodium* (stork’s bill) and *Bunium* type (which includes species such as dill). There is also the possibility that this exceptional pollen assemblage actually represents plants that were brought to the site deliberately in order to enrich the diet. The appearance of both desert sunrose pollen type (*Helianthemum sancti-antonii* type) and desert truffle spore type (*kmehat hanegev*) at some of the Mashabe Sade pollen assemblages also provides evidence for the gathering opportunities which were prevalent near the site. These two species grow in a symbiotic relationship (ectomycorrhizal) in the arid zones of the Mediterranean Basin. The gathering season of the truffle is late winter to early spring.

#### 4.2.4 Haroa

The palynological spectrum of the sample taken from the central courtyard was composed of typical desert elements, and most probably represents the ancient pollen rain (P32). The components of this sample show a clear resemblance to those identified in the recent surface sediment sample (P35). This observation suggests that similar flora existed near the site during the Iron Age II and in modern times. In sample P33 proposed as degraded mudbricks and in sample P34 suggested as dung pollen was not preserved. Within the section composed of fifteen samples collected from the rock shelter, only the six uppermost samples (from the surface to a depth of 50 cm; P36-P50) included well-preserved pollen grains (S1 Fig). These samples are representative of recent and sub-recent times, and therefore can only serve as control samples. In addition to edible plants previously identified in livestock dung, they include members of the aster family and chenopods, wind-pollinated Mediterranean trees such as *Pinus* and *Olea*. This probably stems from the fact that it is an open rock shelter that is probably composed of both degraded livestock dung deposits, aeolian loess sediments and airborne pollen, as was shown for another rock shelter in the region [66]. The presence of plants blooming at different periods of the year supports the observation that the accumulation at Haroa rock shelter represents a mix of wind-transported pollen grains and pollen from degraded dung. The rock shelter samples are characterized by higher pollen concentrations in comparison to the archaeological and control samples (reaching up to 88,890 pollen/g/sediment). Indeed, high pollen concentrations are a characteristic of dung material. Except for four grains that were found in the sub-modern sediments of the rock shelter (P41), cereal pollen was not found in any of the samples analyzed from Haroa. All samples collected from Nahal Mesora rock shelter were pollen barren (P51-P54). This is likely caused by the burning of the dung deposits to conserve space, which is a common practice in the region. The high frequencies of microcharcoals in these spectra corroborate this assumption.

## 5. Discussion

All of the pollen taxa identified in this study from various archaeological contexts (activity surfaces, dung material, rock shelters, etc.) are still characteristics of the Negev Highlands today. The same observation emerged from wood charcoal analyses conducted at Ein Ziq and Intermediate Bronze Be’er Resisim [53, 67]. This indicates that during the peaks of occupation in the Negev Highlands the local environment was not significantly different from what prevails today. Despite the dominant northwest winds (especially during the blooming season, Fig 2C), we found that the contribution of Mediterranean pollen to the research area is negligible (in both the archaeological and the recent control samples). It can therefore be concluded that the pollen found in this study primarily originated in the Negev Highlands and is therefore a good representative of local ancient vegetation.

The palynological analyses of dung deposits retrieved from the Early Bronze and Intermediate Bronze settlement site of Nahal Boqer 66 indicate that livestock there consumed wild taxa. The pollen spectra were composed mainly of plants belonging to Asteraceae Asteroideae (aster-like) pollen type and Chenopodiaceae (goosefoot). It is therefore suggested that the animals were free grazing (pure pastoralism) rather than fed or supplemented with agricultural by-products (agro-pastoralism). Indeed, all Nahal Boqer 66 palynological assemblages, from all periods of occupation, point to a total absence of cereal pollen type. The lack of dendritic phytoliths at the site (which may be indicative of cereal agriculture) [19], corroborates our palynological observation. According to Dunseth et al. [8], human activity at Nahal Boqer 66 spans from the Early Bronze Age IB till the early phase of the Intermediate Bronze Age (ca. 3200–2200 BCE). Hence, there is no archaeobotanical and palynological evidence to support earlier ideas of seasonal or opportunistic dry-farming practiced near settlements of this type [13: p. 73–74; 68–72]. The overlap in the blooming season of the most dominant pollen taxa suggests that the site was occupied only during late winter and spring. This is shown, for instance, in the almost total absence of wormwood (*Artemisia*) and acacia pollen, which bloom during autumn and early winter. The only sample with autumn bloomer taxa did not originate from activity surfaces but from the nearby rock shelter of Umm Urgan (Table 1). Based on a study conducted by Babenko et al. [66], such a palynological spectrum represents a mix of degraded herd dung pollen together with degraded dung from wild animals, as well as the deposition of airborne pollen. This too supports our observation that herding activities at the Negev Highland sites took place only during late winter and spring.

The nearly total absence of cereal pollen from the four Negev Highlands sites studied here, together with the relatively good state of pollen preservation, indicates that the inhabitants of the region did not engage in agricultural activities [supporting 8, 15, 16, 18, 73, contra 74, 75]. In addition to the lack of cereal pollen, the two Intermediate Bronze Age sites, Ein Ziq and Mashabe Sade, are also characterized by the absolute absence of palynological and microarchaeological markers of herding activities [this study; 18]. Nevertheless, one of the Mashabe Sade palynological spectra hints at the potential for plant gathering: pollen of plants such as vetches, croci, stork’s bill and tumble thistle was retrieved from surface activity. The presence of both desert sunrose pollen type and desert truffle spore type which co-exist in the arid zones of the Mediterranean Basin in a symbiotic relationship (ectomycorrhizal), provides evidence for the vegetarian dietary options which were prevalent near the site. In the same way, it was shown by Hakker-Orion [54] that the meat diet of Ein Ziq inhabitants was composed of ca.10% of wild desert animals, including gazelles, birds and hares.

The three earlier sites studied here (Nahal Boqer, Ein Ziq and Mashabe Sade) are representative of the long period of human activity in the southern Levant arid land—throughout the Early Bronze and the first half of the Intermediate Bronze Age (with certain changes in the settlement patterns occurring in the transition from the Early Bronze Age III to the Intermediate Bronze Age; [9]). While the small site of Nahal Boqer 66 was probably occupied by a local pastoral population that was engaged in herding activity, the two central sites of Ein Ziq and Mashabe Sade probably served as trading posts [18, 19]. Trading activity in the Negev Highlands during the third millennium BCE was most likely related to the copper industry in the Arabah and to copper transportation to the neighboring settled lands [9 and references therein]. This may explain the lack of evidence regarding food production at the two sites; they most probably served as short-term parking sites. Demand for copper in Egypt and the accompanying development of trade networks that transported this valuable resource played an important role in the settlement history of the arid regions: the peak of prosperity in the Early Bronze Age III and the first half of the Intermediate Bronze Age corresponds to the time of the 5th and 6th Dynasties in Egypt, and the deterioration of the Negev system accords with the collapse of the Old Kingdom ca. 2200 BCE [9]. Interestingly, a recent study of rock-cut images of “crescent-headed figures” in the Negev has also linked sites along transportation routes from Feinan through the Negev Highlands to north Sinai [76].

Though the Negev Highland settlement pattern is mostly related to the copper industry, climate changes should also be taken into consideration. Specifically, dryness is synchronized with the decline of the Negev settlement system during the second half of the Intermediate Bronze Age. The Negev Highlands area receives most of its rain from the Mediterranean climate system [29, 30]. A dry period that began in ~2200 BCE and lasted about 300 years, is evidenced by palynological records from the Sea of Galilee and the Dead Sea, areas which are also influenced mainly by the Mediterranean climate system [36, 37]. From the historical-geographical point of view, this dry period (also known as the 4.2 ka event) took place across the entire Levant [33, 77 and references therein], in fact across the entire Near East [78], as well as other parts of the Mediterranean Basin [recently reviewed by 79]. This abrupt climate episode radically altered precipitation in the Eastern Mediterranean, with an estimated >30% drop in rainfall [77]. Such prolonged droughts must have had a devastating effect on the vulnerable Negev Highlands settlement system, preventing even seasonal occupation. In contrast, humid conditions prevailed among Negev settlement peaks prior to the 4.2 ka event during the Early Bronze and the first half of the Intermediate Bronze Age [37, 43, 80–83].

The total absence of cereal pollen (this study) and phytolith [15] from the dung context at Iron IIA Haroa (late 10th century-9th century), points to free-grazing pure pastoralism. A recent pollen study on livestock pens identified in the gatehouse of "Slave’s Hill" at Timna (one of the largest copper smelting camps in the Timna Valley, dated to the 10th century BCE; [84]) enabled us to differentiate between the practices of free-grazing and feeding animals with agricultural byproducts as the main component in their diet. As was shown in this study and in a previous pollen study from the same region (Atzmaut Rock shelter, [66]), palynological assemblages of animal dung from free-grazing herds in the Negev Highlands are typified by the occurrence of only wild desert species, as animals primarily feed on nearby local shrub vegetation [15, 66]. The palynological assemblages retrieved from Timna are dominated by cereal pollen (ca. 90% of the total pollen counted in each sample), most likely wheat (*Triticum* pollen type). Some other components within Timna’s pollen samples seem to originate from the Mediterranean zone and penetrated into the assemblages probably from the nearby vegetation which surrounded the fields where the wheat was cultivated (e.g., pine). The study shows that the gatehouse area at Timna was used for storing donkeys (or mules), which were the common draught animal at the time, together with other livestock (probably goats; [84]). The animals were fed with hay (rather than straw as evidenced by the occurrence of pollen grains) that originated from the Mediterranean region (where the minimum of 400 mm of annual rainfall required for wheat cultivation exists), more than 200 km to the north (Philistia/Judea). The fodder was probably brought along the road that connected Timna to Egypt (the main source of copper demand) along the coast. This food reflects special treatment and care, in accordance with the critical role of donkeys in the success of copper transport and trade in a logistically challenging region [84].

A study conducted at Early Islamic Shivta (located in the western Negev), on dung pellets of domestic sheep and goats, clearly demonstrates the ability of archaeobotanical proxies to infer herding practices from dung material. The study is based on a combined archaeobotanical reconstruction (pollen, phytolith and seeds), suggesting that during the mid-7^th^-mid-8^th^ centuries CE, spring-time herding at Shivta was based on free-grazing of wild vegetation, supplemented by chaff and/or hay from domestic cereals. For the late 8^th^-mid-10^th^ century CE samples, phytolith and pollen reconstruction indicate autumn-winter free-grazing with no evidence of foddering [85]. When compared to control samples of surface and subsurface sediments, most palynological assemblages recovered from contexts related both to structures (buildings and courtyards) and to dung had high ratios of pollen clumps from plants belonging to the goosefoot family (Fig 8). In our view, the appearance of these clumps may indicate one or both of the following possibilities: (1) livestock ate the entire chenopod inflorescence (pollen grains are attached in the flower); (2) the clumps may have fallen on the surface activities from flowers on chenopod branches used for roofing.

## 6. Conclusions

This study presents the results of palynological investigations conducted at Bronze and Iron Age sites in the Negev Highlands. The interpretation of the palynological spectra sheds light on the subsistence economy mode and the nature of occupation at these sites. The study also enables us to evaluate the role played by environmental and climatological changes in shaping trends in the Negev Highlands settlement oscillations. The following conclusions can be drawn:

1. While our pollen evidence indicates that the Negev Highlands vegetation during peaks of occupation was similar to that which presently prevails, it is assumed that the wet climate conditions reconstructed in the Mediterranean zone during the Early Bronze and first half of the Intermediate Bronze Age supported the activity in the Negev Highlands (since the area receives most of its rain from Mediterranean climate systems). The lack of settlement activity in the region during the second half of the Intermediate Bronze Age coincides with ca. 300 years of dry climate in the entire Near East, beginning at ca. 2200 BCE.
2. The palynological spectra retrieved from dung deposits indicate that livestock herds during the Intermediate Bronze Age were free grazed on wild vegetation only, and were not supplemented with agricultural by-products. It can therefore be concluded that Negev Highlands inhabitants of that period who herded animals were engaged in a purely pastoral herding strategy and were not involved in an agro-pastoral economic strategy.
3. It also seems that the Negev Highlands sites where herding activities took place, were occupied only seasonally, during late winter and spring, rather than year-round.
4. The lack of cereal pollen type indicates that the occupation in the Negev Highlands sites during the third millennium BCE, as well as during the Iron Age IIA was probably related to the copper industry in the Arabah and to copper transportation to the neighboring settled lands.
5. Our results also hint at the potential for plant gathering in the Negev Highlands based on the presence of edible plant remains.

## Supporting information

S1 Fig Simplified pollen diagram of Haroa rock shelter sequence (from surface and until 50 cm below surface). A 10-fold exaggeration is used to show changes in low taxa percentages.
(PDF)
