# Supplementary material for: Environment, subsistence strategies and settlement seasonality in the Negev Highlands (Israel) during the Bronze and Iron Ages: The palynological evidence
Source: PLoS One. 2023 May 24;18(5):e0285358. doi: 10.1371/journal.pone.0285358 (PMC10208521; doi:10.1371/journal.pone.0285358)
Supplement: S1 Fig — A 10-fold exaggeration is used to show changes in low taxa percentages. (PDF) [file pone.0285358.s001.pdf]

Supplementary Material

Figure S1: Simplified pollen diagram of Haroa rock shelter sequence (from surface and until 50 cm below surface). A 10-fold exaggeration is used to show changes in low taxa percentages.

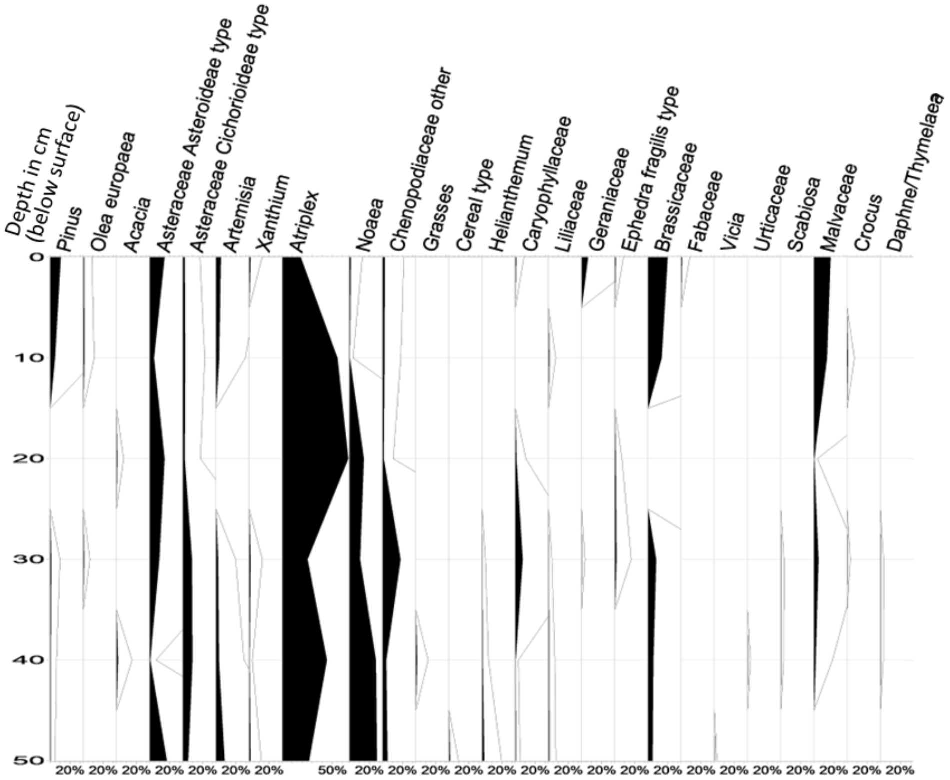

For the preparation of the palynological diagram, PolPal software was used [1].

References

Walanus A, Nalepka D. POLPAL Program for counting pollen grains, diagrams plotting and numerical analysis. Acta Palaeobotanica. 1999;2, 659–661.
